# Neuropeptides and the Autonomic Nervous System in Prader–Willi Syndrome

**DOI:** 10.3390/ijms27010352

**Published:** 2025-12-29

**Authors:** Charlotte Höybye, Maria Petersson

**Affiliations:** 1 Department of Endocrinology, Karolinska University Hospital, 171 76 Stockholm, Sweden; charlotte.hoybye@ki.se; 2 Department of Molecular Medicine and Surgery, Karolinska Institutet, 171 77 Stockholm, Sweden

**Keywords:** Prader–Willi syndrome, autonomic nervous system, neuropeptides

## Abstract

Prader–Willi syndrome (PWS) is a rare, multisymptomatic genetic disorder caused by the absence or dysfunction of specific genes on chromosome 15. The genetic abnormality is anticipated to cause a dysfunction of the hypothalamus, which is also central in the regulation of the autonomic nervous system (ANS). Typical symptoms of PWS indicating a hypothalamic dysfunction include muscular hypotonia, poor growth, short stature, and feeding difficulties in infancy, which in early childhood are replaced by hyperphagia, leading to a high risk of obesity. Other characteristics, such as sleep difficulties, altered pain perception, delayed gastric emptying and constipation, blood pressure irregularities and dysregulated stress response, altered temperature regulation, delayed pupillary reaction, and urine retention and incontinence, all indicate a dysfunction of ANS. The ANS is usually divided into three parts: the sympathetic nervous system (SNS), which activates the fight-or-flight response during stress; the parasympathetic nervous system (PNS), which promotes calm and digestion; and the independent enteric nervous system (ENS), which regulates the gastrointestinal tract. Noradrenaline is the main neurotransmitter for the SNS, and acetylcholine for the PNS, while the ENS is regulated mainly by acetylcholine and serotonin. However, the ENS is modulated by both the SNS and the PNS, as well as many neuropeptides. Peptides regulating behavior, metabolism, appetite, and satiety have been extensively studied in PWS. However, studies of the role of neuropeptides in regulating other autonomic functions are limited and remain poorly understood. This review aims to synthesize current evidence from both animal models and human studies to explore potential mechanisms by which neuropeptides may contribute to autonomic dysfunction in individuals with PWS.

## 1. Introduction

### 1.1. PWS

Prader–Willi syndrome (PWS) is a rare, multisymptomatic disorder. The incidence is one in 10.000–20.000 newborns, and the prevalence is one in 10.000–21.000 individuals [1]. The syndrome is caused by the lack of expression of paternal genes on chromosome 15 in region q11.2–q13, of which 65–75% is caused by paternal deletion, 20–30% by maternal uniparental disomy (mUPD), and 2–5% by imprinting defects [1]. Characteristic symptoms are cognitive dysfunction, neurodevelopmental delay, muscular hypotonia, hormone deficiencies, hyperphagia, and behavioral challenges [2]. Neonates with PWS are extremely hypotonic with poor sucking ability and failure to thrive. In early childhood, this clinical presentation shifts to hyperphagia, leading to a high risk of severe obesity and associated comorbidities, such as type 2 diabetes and heart failure. The genetic alterations result in hypothalamic and pituitary dysfunctions, including growth hormone (GH) deficiency, hypogonadism, and hypothyroidism, whereas central adrenal insufficiency is rare. Several studies have shown that GH treatment improves height, body composition, metabolism, motor function, and cognition, and GH treatment has dramatically changed the syndrome [2]. Nutritional needs are decreased, and a restricted, controlled diet, regular exercise, and hormone replacement, as well as screening for and treatment of comorbidities, are cornerstone treatments [2,3].

The hypothalamus is central in the regulation of the autonomic nervous system (ANS), and efferent and afferent signals within ANS are integrated within the hypothalamus and the brainstem. ANS consists of the sympathetic (SNS) and parasympathetic nervous systems (PNS). Dysregulation of either the SNS or PNS, or both, may contribute to the development of obesity and related metabolic comorbidities [4], and ANS dysfunction has been implicated in various forms of childhood obesity [5,6]. Other symptoms in PWS indicating a dysfunction of ANS include altered temperature regulation with hypo- or hyperthermia, delayed gastric emptying, urine retention and incontinence, reduced heart rate variability, sleep-disordered breathing, decreased salivation, and poor pain perception [7]. In addition, the enteric nervous system (ENS) is often considered the third branch of the ANS. ENS is an independent network of neurons within the gastrointestinal tract, but it is modulated by both the SNS and the PNS, and thus indirectly by the hypothalamus.

### 1.2. The Autonomic Nervous System

The ANS is the part of the nervous system that controls internal organs, smooth muscles, glands, and the gastrointestinal tract, and it operates primarily involuntarily. It regulates vital functions such as respiration, circulation, temperature, digestion, and sleep, and it also participates in metabolism and sexual function. The ANS, and, in particular, the SNS, is responsible for the fight-or-flight response, which is closely linked to the hypothalamic–pituitary–adrenal (HPA) axis, while the PNS is central for growth, digestion, and restorative processes. The PNS and the ENS are closely connected in regulating secretion, peristalsis, and the release of regulatory peptides in the gastrointestinal tract [8].

The activity within the ANS is regulated by neurotransmitters, with acetylcholine being the main neurotransmitter in the PNS and noradrenaline being the main neurotransmitter in the SNS, with adrenaline and dopamine contributing. For the activity in the ENS, serotonin is fundamental. Moreover, neuropeptides influence the activity of all three parts of the ANS [8,9,10]. To explore the possible role of these peptides in symptoms of ANS dysfunction in individuals with PWS, we reviewed the literature, including both animal models of PWS and individuals with PWS.

### 1.3. Peptides Regulating the Autonomic Nervous System

The activity within the ANS is not solely dependent on neurotransmitters. The activity within the ANS is modulated by peptides at all levels, from the hypothalamus and the brainstem to the periphery.

For example, in the hypothalamus, corticotropin-releasing hormone (CRH), which is the main regulator of activity within the HPA axis, also stimulates the activity of the SNS [11]. In contrast, oxytocin (OXT) inhibits the sympathetic nervous system and stimulates the activity within the parasympathetic nervous system, through effects within both the hypothalamus and the brainstem [12,13].

Interestingly, CRH and OXT are both produced within the same hypothalamic nucleus, the paraventricular nucleus (PVN), and they modulate each other’s release [14]. In the PVN, arginine vasopressin (AVP) and thyreotropin-releasing hormone (TRH) are also produced. TRH is the main regulator of the pituitary–thyroid axis and is also an important modulator of the activity within the ANS. Furthermore, TRH is of central importance for thermoregulation [15].

In the brainstem, OXT, AVP, CRH, and TRH, as well as, for example, galanin, substance P, and neuropeptide Y (NPY) modulate the activity within the nucleus of the solitary tract (NTS) and the dorsal vagal motor nucleus (DMX) [16]. NTS and DMX are of fundamental importance for the integration of afferent and efferent signals within the ANS. AVP and vasoactive intestinal peptide (VIP) are significant for diurnal rhythm and sleep and are even synthesized within the suprachiasmatic nucleus itself (the nucleus of key importance for circadian rhythms) [17]. Another important area is the rostral ventrolateral medulla (RVLM), where, for example, OXT, CRH, as well as neuropeptide Y (NPY) and AVP, are important modulators of sympathetic activity and blood pressure regulation [18].

All of the above-mentioned peptides also modulate the activity in the autonomic ganglia and peripheral nerves. In the periphery, VIP regulates, for example, blood vessels, peristalsis, and the secretion of bile and pancreatic juice, as well as electrolyte and water excretion in the gastrointestinal tract [19]. Cholecystokinin (CCK) is another peptide of great importance here, as well as ghrelin, which, besides stimulating hunger, also affects gastrointestinal motility and vagal tone [20,21]. In contrast, somatostatin inhibits all these effects as well as the release of most other peptides and hormones.

Additionally, peptides such as calcitonin gene-related peptide (CGRP), substance P, galanin, and orexin are important modulators of the ANS, in particular, for nociception, temperature regulation, and diurnal rhythm regulation [9,10].

Several of these peptides are likely implicated in the pathophysiology of PWS, contributing to symptoms such as altered pain sensitivity, temperature, and cardiovascular dysregulation, sleep disturbances, and gastrointestinal dysfunction.

Examples of modulating peptides in relation to symptoms and dysfunctions in PWS are shown in Table 1.

## 2. Deviations of Peptides Regulating the Autonomic Nervous System in PWS

Studies on peptides in individuals with PWS have predominantly focused on regulation of appetite and satiety, metabolic control, and behavior (see, for example, review [22,23]), and, consequently, neuropeptides such as OXT, ghrelin, and glucagon-like peptide-1 (GLP-1) have been extensively investigated in this context. However, the study cohorts were small, and the findings are not always consistent. In contrast, studies of their roles for ANS dysfunction in other aspects and research on other peptides with potential relevance to ANS regulation, such as somatostatin, VIP, and substance P, remain limited or entirely lacking. In the following section, we explore the possible involvement of different neuropeptides in ANS dysfunction beyond metabolism, behavior, and appetite. Importantly, concentrations of peptides are not always congruent in, for example, blood, cerebrospinal fluid, and brain, and differences in the effects might, of course, also depend upon receptor function, such as receptor number and affinity, as well as second messengers and intracellular mechanisms.

### 2.1. Oxytocin and Arginine Vasopressin

OXT and AVP share evolutionary origins and differ by only two of the nine amino acids. Both can act as neurotransmitters, neuromodulators, and hormones (see, for example, [24]). They are produced within the PVN and supraoptic nucleus (SON) within the hypothalamus. Besides their well-known hormonal effects, i.e., uterine contraction, milk ejection, and antidiuresis, respectively, they modulate the activities in the ANS and the HPA axis. When released in high amounts or administered in high doses, they can interact with each other’s receptors, but during normal physiological conditions, the receptor selectivity is largely maintained. OXT and AVP receptors are widespread both in the brain, brainstem, and in the periphery, for example, in the liver, the gastrointestinal tract, the endocrine part of the pancreas, and in the muscles, as well as in the adipocytes [25,26,27,28,29].

#### 2.1.1. Oxytocin

OXT has multiple behavioral and metabolic effects. It interacts with several other neuropeptides and hormones of importance and modulates several of the effects listed in Table 1. OXT enhances PNS activity and decreases the activity within the HPA axis [30]. It participates in the regulation of cardiovascular function through both direct and indirect effects and increases the activity of alpha 2-adrenoreceptors, which also contributes to the antinociceptive effects caused by OXT [31,32]. This effect has also been shown to involve an increase in endogenous opioids [33]. In addition, OXT has been shown to modulate temperature and diurnal rhythm. For example, OXT knockout mice exhibit dysregulation of temperature, which can be restored if OXT receptors are activated [34]. Recent studies also demonstrate that OXT participates in diurnal thermoregulation [35].

Magel2 is one of the paternally expressed genes in the 15q11-q13 region. Magel2-knockout mice are commonly used as a model in the studies of PWS, and these mice have decreased OXT levels. Another knockout mouse model used as a model for PWS is the Necdin-knockout mouse, which has reduced OXT levels as well. Both mouse models have disrupted respiratory patterns and alterations in circadian rhythm [36]. Additionally, the Necdin knockouts have increased nociceptive thresholds [37]. Of course, these mouse models cannot exactly be compared to individuals with PWS, but they have some similar features, and, therefore, comparisons are interesting.

In humans, studies of postmortem brain tissue from adults with PWS have shown a 42% decrease in the number and a 54% decrease in the volume of OXT-expressing neurons in PVN, suggesting OXT to be the cause for hyperphagia in PWS and contributing to the effects listed above [38,39]. Moreover, very low concentrations of OXT in lymphoblastoid cells and in postmortem frontal cortex tissue have been found [40]. Based on these findings, decreased levels of plasma OXT would be expected, but in contrast, increased OXT levels in PWS have been reported. In a study of 23 children with PWS, plasma OXT levels were compared with 18 healthy, unrelated siblings matched for age and with a similar gender ratio and BMI [41]. The authors found that children with PWS had more than twice as high levels of plasma OXT as compared to unrelated siblings [41]. In 17 adults with PWS (nine men and eight women), similar concentrations of serum OXT as in controls were observed, but, in relation to obesity, the concentrations were low [42]. Additionally, OXT in cerebrospinal fluid (CSF) in two men and three females with PWS was higher than in the controls [43]. However, important to note, central levels and plasma levels of neuropeptides are not always in parallel.

#### 2.1.2. Arginine Vasopressin (AVP)

Like OXT, AVP also contributes to the modulation of ANS and HPA axis activity. It plays a role in cardiovascular regulation through both central and peripheral mechanisms, with AVP itself exerting a strong vasoconstrictive effect through AVP V1a receptors. Moreover, AVP is crucial for maintaining diurnal rhythm, acting as a key regulator within the suprachiasmatic nucleus—commonly referred to as “the body’s clock [44].” Magel2-knockout mice appear to exhibit irregular activity in this nucleus, which may involve AVP-related mechanisms. In addition, AVP levels in the lateral septum of Magel2-knockout mice are reduced. Restoration of vasopressin signaling in this region has been shown to improve social deficits in these animals [45].

Studies have examined AVP levels in individuals with PWS. Swaab et al. [38] reported a comparable number of hypothalamic AVP-producing neurons in individuals with PWS compared to controls. In contrast, Martin et al. found reduced CSF AVP levels in three females with PWS compared to female controls [43]. Additionally, lower plasma AVP concentrations have been reported in a study with 30 adolescents and adults [46], although the same study noted that within the PWS maternal uniparental disomy (mUPD) subtype, higher plasma AVP levels were associated with increased behavioral problems. In a follow-up study of the same cohorts, those with mUPD had higher plasma AVP, which was associated with lower respiratory sinus arrhythmia (RSA) and heart rate variability, compared to controls [47]. These findings suggest a dysfunction in the ANS in PWS, more pronounced in the mUPD subtype, indicating greater loss of parasympathetic activity. Supporting the notion of AVP dysregulation in PWS, the neuroendocrine chaperone protein 7B2—which facilitates the activation of prohormone convertase 2 (PC2) and thereby the processing of vasopressin precursors—was undetectable in two out of five PWS patients [48]. Further evidence comes from magnetic resonance imaging studies showing that the signal intensity of the “bright spot” in the posterior pituitary gland was negatively correlated with hyperphagia and ASD-like behaviors in adolescents and adults with PWS [49].

### 2.2. Neuropeptide Y (NPY)

NPY, together with agouti-related peptide (AgRP), is located within the arcuate nucleus, where they act to stimulate appetite. However, NPY is also produced in many other nuclei and in the periphery. NPY is of central importance for thermoregulation, nociception, and diurnal rhythm, and it is a major autonomic modulator often collocated with noradrenaline in the sympathetic nerves, for example, in those mediating vasoconstriction, pupillary reaction, and salivation [50,51]. NPY also increases sympathetic tone to the urinary bladder, enhancing detrusor contraction, which may have a role in urine retention [52].

Thus, although potentially relevant, direct evidence of NPY acting in ANS dysfunction in PWS is limited. Since NPY stimulates hunger, it has been expected that centrally located NPY neurons would be upregulated. But instead, the numbers have been demonstrated to be downregulated [23] or unchanged [53]. In a study of plasma NPY levels in adults with PWS, it was shown that NPY levels were within the lower normal range (despite obesity) and did not significantly change with GH treatment [42].

However, given the role of NPY in enhancing sympathetic nervous tone, an altered NPY signaling could hypothetically contribute to dysregulated autonomic function in PWS, such as impaired thermoregulation, altered pupillary reactions, and cardiovascular regulation, as well as urinary dysfunction.

### 2.3. Ghrelin

Ghrelin is an orexigenic hormone mainly produced by enteroendocrine cells in the gastrointestinal tract [54,55], but ghrelin is also produced within the brain, for example, in the hypothalamus. Ghrelin receptors are located both in the brain and in the periphery, and it is a potent stimulator of GH secretion [20,56].

Administration of ghrelin potentiates hunger and feeding behavior, and ghrelin levels are elevated in fasted conditions [57]. Besides these effects, ghrelin has been demonstrated to increase gastric emptying and peristalsis, and a dysfunction in ghrelin receptors has been suggested to contribute to constipation [58].

It is well-established that plasma ghrelin levels are elevated in PWS [59,60], but a direct change in the orexigenic effect of ghrelin, within its physiological range, has not been demonstrated. One study reported delayed gastric emptying in PWS, despite the presence of higher ghrelin levels, which, as mentioned above, generally promote gastric emptying [61]. Although ghrelin is primarily known as an appetite-regulating hormone, it may affect both afferent and efferent vagal nerve activity, thus affecting gastric motility and possibly other autonomic functions. However, Magel2-knockouts, which, of course, do not always mirror individuals with PWS, appear to have unchanged ghrelin levels [62].

### 2.4. Cholecystokinin (CCK)

CCK is produced both in the brain and in the gastrointestinal tract. It is a key mediator of vagal afferent signaling, particularly in the regulation of satiety [63], as well as in the modulation of gastrointestinal motility and the release of other peptides. CCK receptors are expressed in both central and peripheral tissues.

CCK coordinates the digestive process through endocrine, paracrine, and neurocrine mechanisms by stimulating pancreatic and gallbladder secretions, inhibiting gastric emptying, and modulating intestinal motility. In addition, CCK has been shown to enhance nociception and to interact with other signaling molecules such as opioids and substance P [21].

One study found similar fasting CCK levels in adults with PWS compared to obese controls, but the correlation between free fatty acids and CCK seen in controls was absent in PWS [64]. Other studies reported elevated mean CCK levels in PWS, yet no postprandial increase following meals [65,66]. Together, these findings suggest that although basal CCK levels may be normal or even elevated, impaired dynamic regulation contributes to dysregulated CCK-mediated satiety signaling in PWS.

### 2.5. Substance P

Substance P, as well as its receptors, is present both in the brain and within the periphery. It is of particular importance for nociception and inflammation. Additionally, substance P is also involved in the regulation of ANS activity within the brainstem, and it contributes to cardiovascular and respiratory regulation as well as gastric motility. In the periphery, it promotes vasodilatation and is present in, for example, sensory nerves, enhancing nociceptive signals as well as regulating the eccrine sweat gland function [67,68,69].

Substance P has been sparsely studied in PWS. However, in 23 children with PWS (5–11 years), plasma levels of substance P were higher than in unrelated, unaffected siblings after adjusting for age, sex, and BMI [70].

### 2.6. Glucagon-like Peptide-1 (GLP-1)

Glucagon-like peptide-1 (GLP-1) is primarily synthesized by L-cells in the duodenum, small intestine, and, in smaller quantities, by the pancreas. However, production has also been demonstrated in the brain and the brainstem [71].

Its secretion in the gastrointestinal tract is modulated by glucose and fatty acid levels following food intake or stimulation of the vagus nerve. The main mechanisms of action of GLP-1 include stimulation of insulin secretion by beta-cells in the islets of Langerhans and inhibition of glucagon secretion by alpha-cells. GLP-1 exerts its central effects through the GLP-1 receptor in the central nervous system, reducing the rate of absorption of food into the blood via appetite suppression and delayed gastric emptying [71,72,73]. Recent studies have also demonstrated that GLP-1 can modulate cardiovascular regulation and stress responses via the HPA axis [74].

Fasting GLP-1 concentrations in adults with PWS have been found to be similar to individuals with obesity and lean controls [75,76]. However, other studies found that fasting GLP-1 concentrations in adults with PWS were higher than in the obese and lean controls [77]. GLP-1 receptor-agonists are used in patients with PWS for weight, glycemic, and appetite control [78]. However, systematic studies are lacking. Effects and side effects have not been completely described, and more studies are needed to define the use of GLP1 receptor agonists in PWS.

### 2.7. Orexin

Orexin is mainly produced within neurons in the hypothalamus, and, besides its role in metabolism, it is important for the diurnal rhythm [79]. Orexin has even been suggested to be involved in the pathophysiology of narcolepsy, and it has a central role in pupillary reactions [80].

Orexin is an important modulator of the ANS affecting respiratory and cardiovascular function, gastrointestinal motility, as well as nociception and temperature regulation [81,82].

Orexin has been measured in 23 children with PWS and was found to be higher in plasma compared to controls [83]. For comparison, Magel2 knockouts have reduced orexin levels as well as reduced orexin-positive neurons within the hypothalamus [84].

### 2.8. Opioids

Endogenous opioids, endorphins, enkephalins, dynorphins, and the lesser-known endomorphins are neuropeptides produced in both the brain and the peripheral nervous system.

These peptides modulate autonomic functions by acting on three different receptors: μ-, δ-, and κ-opioid receptors. For example, β-endorphin can suppress sympathetic outflow and reduce heart rate and blood pressure. Enkephalins, located in the spinal cord and enteric neurons, inhibit gastrointestinal motility and secretion. Dynorphins, produced in the brainstem and limbic regions, influence stress responses and thermoregulation [85,86].

To our knowledge, there is only one published study of the levels of endogenous opioids in PWS. In the same study where substance P was measured (see above), beta-endorphin levels were also assessed and were found to be elevated in children with PWS [70].

### 2.9. Thyreotropin-Releasing Hormone (TRH) and Corticotropin-Releasing Hormone (CRH)

TRH is the main regulator of the pituitary–thyroid axis, and CRH is the main regulator of the HPA axis. They are both mainly produced within the PVN and modulators of, for example, temperature, diurnal rhythm, nociception, and cardiovascular regulation [11,15]. CRH increases the activity within the SNS, whereas TRH most often, but not always, promotes PNS activity [87,88]. CRH has been implicated in stress-induced gastrointestinal dysmotility via CRH receptors in the enteric nervous system [89], which might contribute to the high prevalence of constipation and delayed gastric emptying in individuals with PWS.

Additionally, both TRH and CRH interact with monoaminergic systems such as serotonin and norepinephrine, which are critical for autonomic tone and stress reactivity. Altered TRH signaling may also affect thermoregulation and vagal tone, potentially contributing to the blunted autonomic responses observed in PWS [11,15,87,88].

Indeed, a dysregulation of the HPA axis with a delayed stress response has been suggested in PWS [90]. Magel2-null mice had elevated basal corticosterone levels, and although male Magel2-null mice had an intact corticosterone response to restraint and to insulin-induced hypoglycemia, female Magel2-null mice failed to respond to hypoglycemia with increased corticosterone [91]. 

Findings across studies of a possible dysregulation of the thyroid axis in PWS have been mixed. While the study by Pellikaan et al. [92] reported a higher prevalence of hypothyroidism among adults with PWS, other investigations have not consistently supported a clear disruption of pituitary–thyroid axis function [93].

### 2.10. Other Neuropeptides of Importance for the Modulation of ANS Activity

In addition to the neuropeptides discussed above, several others are known to participate in the modulation of ANS activity. For example, galanin, VIP, and calcitonin gene-related peptide (CGRP) are all produced in the brain, brainstem, and peripheral tissues.

Galanin modulates cardiovascular function, nociception, and gastrointestinal motility. There are three types of galanin receptors, which may exert opposing effects depending on receptor subtype and tissue context. For instance, galanin can have contrasting modulatory roles in thermoregulation depending on which receptor is activated [94,95].

In the brain, VIP contributes to the regulation of circadian rhythm. It is co-localized with acetylcholine in parasympathetic and enteric nerve fibers, where it mediates smooth muscle relaxation and vasodilation. VIP is a key peptide in ANS regulation and can enhance parasympathetic activity. In the gastrointestinal tract, VIP promotes vasodilation, enhances peristalsis, and stimulates the secretion of water and electrolytes. In certain states of autonomic dysfunction, such as diabetic neuropathy, disturbed VIP release has been demonstrated, leading to uncoordinated peristalsis and constipation [19,96]. In the urogenital tract, VIP is released from parasympathetic nerve endings, relaxing the urinary bladder, increasing urinary flow, and decreasing resistance during bladder emptying [97]. However, VIP is also released from cholinergic sympathetic nerve fibers that innervate sweat glands, where it stimulates fluid and electrolyte secretion, contributing to increased sweat production and salivation [69]. The Magel-2 knockout mice, often used as a model of PWS as discussed above, have decreased levels of VIP as well [98].

CGRP is an important modulator of nociception and temperature homeostasis. The latter effect is mediated both centrally and via its potent vasodilatory action. Recent studies also suggest that CGRP may play a role in thermogenesis in brown adipose tissue [99,100].

In addition to these three peptides, somatostatin is also of interest for the ANS. Somatostatin is produced both in the brain and in peripheral tissues, with large amounts secreted in the gastrointestinal tract. Somatostatin inhibits secretion, gastrointestinal motility, the release of other hormones, and parasympathetic activity [101].

To our knowledge, no studies have investigated galanin, VIP, or CGRP in individuals with PWS. One study involving somatostatin infusion in adults with PWS showed that somatostatin reduced ghrelin levels without affecting appetite [102], but this appears to be the only published data. Whether somatostatin levels are altered in individuals with PWS remains unknown.

## 3. Vagus Nerve Stimulation (VNS)

An increase in vagal tone activates the PNS, and vagus nerve stimulation (VNS) is a neuromodulation technology administered through either invasive or non-invasive (transcutaneous VNS, t-VNS) approaches and is approved for treatment of several conditions [103,104]. An increased vagal tone changes the neurotransmitters noradrenaline, acetylcholine, and gamma-aminobutyric acid (GABA) [105]. Reduced GABA levels have been observed in individuals with PWS with emotional and behavioral difficulties, including temper outbursts [106]. In patients with seizures, GABA levels increase after stimulation with t-VNS [107,108]. Although it could be discussed if behavioral changes are influenced by the ANS, it is noteworthy that in an open trial of five patients with PWS using four hours of t-VNS daily for 12 months, followed by one month of daily t-VNS for two hours, four patients had a significant reduction in the number and severity of temper outburst after approximately nine months [109]. Notably, using t-VNS for two hours was associated with an increased number of outbursts for all participants [109]. A follow-up study on cardiac markers of circadian vagal activity showed an increase in the rhythm-adjusted mean of heart rate variability, while the rhythm-adjusted heart rate decreased, indicating an increased cardiac vagal activity. In addition, higher rhythm-adjusted mean heart rate variability predicted a lower frequency of emotional outbursts [110].

An increase in GABA and heart rate variability is associated with an increase in PNS activity (increased vagal tone). OXT, NPY, and somatostatin are examples of neuropeptides that take part in the modulation of GABAergic activity. For example, OXT has been found to increase both parasympathetic activity and GABA activity [111,112]. Thus, OXT deficiency might be one contributing factor to the lower GABA levels correlated to behavioral difficulties in PWS. Indeed, vagal stimulation has been demonstrated to increase OXT levels as well as those of several of the other neuropeptides. For example, NPY, which, as mentioned above, also may influence GABAergic transmission and heart rate variability [113]. It is worth mentioning that there are studies of vagal stimulation in other patient groups showing an improvement of gastrointestinal symptoms and a possible role in inflammation [114].

## 4. Discussion

In this review, we have shown that several peptides involved in the regulation of ANS appear to have altered levels or secretion patterns in PWS. The symptoms of PWS are characterized by a combination of metabolic, endocrine, behavioral, and autonomic disturbances. As a hypothalamic disorder, it is plausible that neuropeptides produced within the hypothalamus—including classical hypothalamic-releasing hormones—are dysregulated. These peptides play key roles in regulating the ANS activity, and the interplay between neuropeptide signaling and autonomic output is likely bidirectional. The pathophysiological mechanisms underlying PWS are multifaceted and not determined by a single peptide or a small subset of responsible peptides. The neuropeptides modulate each other’s synthesis, release, and downstream effects, and are themselves regulated by autonomic feedback loops.

As one illustrative example, the diminished effect of CCK on satiety in individuals with PWS may be influenced by elevated ghrelin levels, which in turn may result from dysregulated insulin, leptin, somatostatin, and altered parasympathetic activity. Additional factors, such as reduced GLP-1, increased NPY expression, as well as the hypothalamic dysfunction, per se, may further contribute. Moreover, some studies have reported lower AVP levels, and AVP correlates with some measures of autonomic function in PWS, suggesting lower autonomic (parasympathetic) control, possibly mediated or reflected by AVP deviations. Furthermore, OXT levels also appear to be altered, and like AVP, OXT levels correlate with autonomic function. In addition, oxytocin modulates CCK levels [115], thereby returning to the peptide initially discussed and underscoring the intricate interplay among these regulatory peptides. Similarly, as discussed by Butler and Bittel [116], imbalances between different peptide systems may further contribute to dysregulation of the autonomic nervous system. In addition, in this review, only changes in peptide levels have been discussed, and potential changes in receptor function, enzyme activities, distribution, and modification of peptides into other forms or fragments have not been included. Recent work has emphasized that neuropeptide receptors act as modulators of autonomic circuitry. For example, Casello et al. [117] highlight how receptors for neuropeptides integrate excitatory and inhibitory signaling to shape network activity and autonomic output. Thus, receptor function and circuit-level modulation represent additional dimensions that may be particularly relevant in PWS. A relatively new mechanism contributing to differences in receptor function is DNA methylation of receptor genes. For example, methylation of the oxytocin receptor gene may alter receptor availability and downstream signaling, thereby adding another layer of complexity to neuropeptide system modulation [118].

A variety of genetically engineered mouse models have been employed to investigate the pathophysiology of PWS, as reviewed by Kummerfeld et al. [36]. Among these, the Magel2-knockout mouse is particularly noteworthy due to its phenotypic similarity to several core features of PWS. Magel2-deficient mice exhibit reduced body weight during early development, followed by excessive weight gain in adulthood, accompanied by insulin and leptin resistance. Respiratory abnormalities, including heightened susceptibility to hypercapnic apnea, have also been reported in these mouse models [36,98]. Furthermore, these animals display decreased levels of key neuropeptides such as OXT, orexin, and AVP—peptides associated with anxiety-like behavior, altered energy homeostasis, and autonomic dysfunction [36,98]. Of note, human mutations in Magel2 also cause Schaaf–Yang syndrome (SYS), which shares several clinical features with PWS. While infants with SYS have hypotonia, severe early obesity is not typical. However, adults with SYS often develop hyperphagia and weight gain, resembling PWS. Both syndromes also include autonomic dysfunction, thereby underscoring the translational relevance of Magel2 models [119].

Similarly, Necdin-knockout mice show reduced OXT levels, disrupted respiratory patterns, diminished pain sensitivity, and alterations in circadian rhythm [120]. Combined deletion of Magel2 and Necdin (NdN-Magel2 double knockouts) results in a more pronounced phenotype, including exacerbated sleep-disordered breathing and apneic episodes [121].

Indeed, OXT treatment of Magel2-knockout mice has been demonstrated to alleviate some behavioral characteristics [122]. In humans, intranasal administration of OXT in infants with PWS was shown to improve oral and social skills [123]. While these abilities are not direct indicators of autonomic dysfunction, they may nevertheless reflect altered oxytocin signaling.

Changes in OXT and orexin levels have been demonstrated in individuals with PWS. Deviations in other neuropeptides—including AVP, substance P, ghrelin, CCK, and GLP-1—have also been reported in some studies. However, some results are conflicting, and data on galanin, VIP, CGRP, endogenous opioids, CRH, and TRH in PWS remain scarce or absent. In addition, effects may vary according to age and sex, as well as factors such as diet, fasting status, weight, and activity, among many others. Indeed, studies have reported inconsistency and variability in ANS function among individuals with PWS [124].

Several additional peptides, not discussed in detail here due to their primary association with appetite and metabolism, may nonetheless influence autonomic function. These include α-MSH, proopiomelanocortin (POMC), irisin, agouti-related peptide (AgRP), and leptin. Although leptin is not typically classified as a neuropeptide, small amounts appear to be produced in the brain. Deviations in the function of the neuronal populations involving neuropeptides in the hypothalamus, such as POMC/CART and NPY/AgRP in the arcuate nucleus, are likely involved in the hyperphagic phenotype observed in PWS [22].

To further elucidate the role of neuropeptides in PWS, direct measurements of key ANS-related peptides (for example, VIP, CRH, TRH, substance P (beyond studies of pain), galanin, etc.) in relation to autonomic features are needed. Causal or interventional studies—such as peptide administration with concurrent monitoring of autonomic parameters (e.g., sudomotor function, pupillary reflexes)—would be particularly informative. Comparative studies across PWS subtypes (e.g., deletion vs. maternal uniparental disomy) and assessments of cerebrospinal fluid (CSF) peptide levels or central peptide expression in relation to autonomic function, although challenging in clinical settings, could provide valuable insights. Further investigations using animal models may help clarify these mechanisms. However, the mechanism behind the peptide deviations found in the mouse models used in the studies of PWS, for example, the Magel2-knockout mice, is not fully known.

It would also be of interest to further explore VNS in the context of autonomic regulation in PWS. VNS is known to trigger the release of several peptides, as discussed earlier in this paper, including OXT, which is also released in response to touch and massage [125]. Studies support a role for OXT in the symptomatology of PWS. To our knowledge, no studies have specifically examined the effects of massage therapy in individuals with PWS, which would be very interesting. Another interesting and unexplored area is potential alterations in the ENS in individuals with PWS. To date, no studies seem to have addressed this question.

In conclusion, multiple neuropeptides appear to be dysregulated in PWS and may contribute to the observed autonomic dysfunction (see Table 2). Studies on the function of ANS, in particular studies on ENS, are scarce, and many statements rely on extrapolation from other conditions or general physiology. While further studies are warranted, it will likely remain challenging to delineate the specific roles of individual peptides, given their region-specific expression patterns in the brain and periphery. Moreover, as discussed above, these peptides interact with and modulate each other’s release and downstream effects. Peptide activity may also vary across developmental stages—such as childhood versus adulthood—and fluctuate in response to physiological states, including stress, satiety, and sleep-wake cycles.

## Figures and Tables

**Table 1:** Symptoms of autonomic nervous system (ANS) dysfunction in Prader–Willi syndrome (PWS) and examples of important modulating peptides.

Symptoms	Examples of Possible Peptides Involved
Altered temperature regulation	TRH, CGRP, orexin, oxytocin, NPY
Sweating abnormalities	VIP, substance P, CGRP
Changes in diurnal rhythm	CRH, TRH, AVP, orexin, NPY, VIP
Increased nociceptive threshold	Opioids, Substance P, OXT, galanin, CGRP, CCK, NPY
Altered blood pressure regulation	NPY, CRH, OXT, AVP, VIP
Heart rate variability	NPY, VIP, CRH, OXT, galanin, AVP
Prolonged pupillary reaction	NPY, VIP, Substance P, orexin, CRH
Decreased saliva production	VIP, NPY
Urine retention	NPY. VIP, substance P
Delayed gastric emptying	Ghrelin, CCK, somatostatin, GLP-1
Constipation	Ghrelin, VIP, somatostatin

Abbreviations: AVP = Arginine Vasopressin, CCK = Cholecystokinin, CGRP = Calcitonin Gene-Related Peptide, CRH = Corticotropin-Releasing Hormone, GLP-1 = Glucagon-Like peptide-1, NPY = Neuropeptide Y, OXT = Oxytocin, TRH = Thyreotropin-Releasing Hormone, VIP = Vasoactive Intestinal Peptide.

**Table 2:** Neuropeptides shown to be altered in Prader–Willi syndrome (PWS) or PWS animal model.

Peptide	Change	Human/Animal	References
Oxytocin	Both increased and decreased	Humans: Increase (decrease in relation to obesity) in plasma and CSF. Decrease in CNS tissue. Animal model: Decreased	[36,37,38,39,40,41,42,43]
Vasopressin	Decreased	Humans: CSF and plasma but unchanged in CNS Animal model	[38,43,46,47,48]
Neuropeptide Y	Decreased or unchanged	Humans: CNS and plasma	[23,42,53]
Ghrelin	Increased or unchanged	Human plasma: Increased Animal model: Unchanged	[59,60,62,76]
Cholecystokinin	Decreased or unchanged	Human plasma	[65,66]
Substance P	Increased	Human plasma	[70]
GLP-1	Increased or unchanged	Human plasma	[75,76,77,78]
Orexin	Both increased and decreased	Human: plasma: Increased Animal model: Decreased	[83,84]
Beta-endorphin	Increased	Human plasma	[70]
VIP	Decreased	Animal model	[98]

Abbreviations: CSF = Cerebrospinal Fluid, CNS = Central Nervous System, GLP-1 = Glucagon-like peptide-1, VIP = Vasoactive Intestinal Peptide.

## Data Availability

This article is a review and does not report new data. Data sharing is therefore not applicable.
